# Artificial Intelligence for Smoking Cessation in Pregnancy

**DOI:** 10.7759/cureus.63732

**Published:** 2024-07-03

**Authors:** Vasiliki E Georgakopoulou, Athina Diamanti

**Affiliations:** 1 Department of Pathophysiology/Pulmonology, Laiko General Hospital, Athens, GRC; 2 Department of Midwifery, University of West Attica, Athens, GRC

**Keywords:** predictive analytics, maternal health, pregnancy, smoking cessation, artificial intelligence

## Abstract

Artificial intelligence (AI) has emerged as a revolutionary tool in various healthcare domains, including smoking cessation among pregnant women. Smoking during pregnancy is a significant public health concern, linked to adverse maternal and fetal outcomes. Traditional cessation methods have had limited success, necessitating innovative approaches. AI offers personalized interventions, predictive analytics, and real-time support, enhancing the effectiveness of smoking cessation programs. This editorial explores the potential of AI in transforming smoking cessation efforts for pregnant women, highlighting its benefits, challenges, and future prospects. By integrating AI into healthcare strategies, we can improve maternal and fetal health outcomes and contribute to the broader public health goal of reducing smoking rates among expectant mothers.

## Editorial

Smoking during pregnancy remains a pervasive public health challenge, with significant implications for both maternal and fetal health. Despite concerted efforts through traditional cessation programs, the success rates have been underwhelming [1].

As technology advances, artificial intelligence (AI) presents a promising avenue for enhancing smoking cessation strategies among pregnant women. AI's potential in healthcare is vast, with its applications ranging from predictive analytics to personalized interventions. For pregnant women struggling to quit smoking, AI can offer tailored support based on individual risk factors and behavior patterns. For instance, machine learning algorithms can analyze a pregnant woman's smoking habits, stress levels, and social support systems to predict relapse risk and recommend timely interventions. Such personalized approaches are likely to be more effective than generic cessation programs, which often fail to address the unique challenges and triggers that pregnant women face. Traditional programs typically offer a one-size-fits-all approach, lacking the flexibility to adapt to individual circumstances such as high stress levels, lack of social support, or specific health conditions that can complicate cessation efforts [2]. 

One of the significant advantages of AI is its ability to provide real-time support. Mobile applications powered by AI can deliver instant feedback, motivational messages, and coping strategies to expectant mothers whenever they feel the urge to smoke. These applications can also connect users to virtual support groups, fostering a sense of community and shared experience, which is crucial for sustaining long-term behavioral change [2].

The future of AI in smoking cessation for pregnant women looks promising, with ongoing research and technological advancements paving the way for more sophisticated and effective interventions. As AI technologies become more accessible and user-friendly, their integration into public health strategies could significantly reduce smoking rates among expectant mothers, ultimately improving maternal and fetal health outcomes [3]. Moreover, AI can enhance the precision of healthcare professionals' efforts. By integrating AI tools into prenatal care, obstetricians can receive detailed insights into their patients' smoking behaviors and the effectiveness of prescribed interventions. This information can help tailor consultations and follow-ups, ensuring that the support provided is aligned with the patient's unique needs and circumstances [4].

However, the integration of AI into smoking cessation programs for pregnant women is not without challenges. Data privacy concerns are paramount, as sensitive information about patients' habits and health must be securely managed. This can be addressed through several measures. First, employing advanced encryption techniques to protect data both in transit and at rest ensures that sensitive information remains confidential. Second, implementing robust access controls limits data access to authorized personnel only. Third, regular security audits and compliance with health data protection regulations, such as the Health Insurance Portability and Accountability Act (HIPAA) in the United States, help maintain high standards of data security. Additionally, educating healthcare providers and users about data privacy and security practices further strengthens the protection of sensitive information [5]. Healthcare providers should also be trained to interpret AI-driven insights correctly and to integrate them into patient care effectively [5].

In conclusion, AI holds great potential in transforming smoking cessation efforts for pregnant women. By providing personalized, real-time support and enhancing healthcare professionals' precision through real-time monitoring and tailored approaches, AI can address the limitations of traditional cessation programs. Despite the challenges, the integration of AI into prenatal care represents a significant step forward in promoting maternal and fetal health. Continued research and collaboration among technologists, healthcare providers, and policymakers will be crucial in realizing the full potential of AI in this critical area of public health.
